# The complete mitochondrial genome of the pentastomid *Armillifer grandis* (Pentastomida) from the Democratic Republic of Congo

**DOI:** 10.1080/23802359.2017.1325341

**Published:** 2017-05-16

**Authors:** José Horacio Grau, Jason A. Dunlop, Martin Meixner, Dennis Tappe

**Affiliations:** a Museum für Naturkunde Berlin, Leibniz-Institut für Evolutions- und Biodiversitätsforschung, Berlin, Germany;; b SMB Services in Molecular Biology GmbH, Ruedersdorf, Germany;; c Nationales Referenzzentrum für tropische Infektionserreger, Bernhard-Nocht-Institut für Tropenmedizin, Hamburg, Germany

**Keywords:** *Armillifer grandis*, tongue worm, pentastomid, parasite

## Abstract

We present the first complete mitochondrial genome of the pentastomid *Armillifer grandis* (Arthropoda: Pentastomida) collected from the lungs of a rhinoceros viper (*Bitis nasicornis*) in the Democratic Republic of Congo. The full length mitochondrial genome of *Armillifer grandis*, which measures 16,073 bp in length, contains 13 protein-coding genes, 2 ribosomal RNA genes, and 22 transfer RNA genes. A clear A + T bias is observed in the mitogenome of *Armillifer grandis* with an overall base composition of 34.6% A, 29.4% T, 29% C, and 6.9% G, and a GC content of 35.9%. The gene arrangement is identical to that of previously described pentastomid mitogenomes.

Pentastomida, colloquially referred to as tongue worms, are an intriguing group of highly adapted vermiform parasitic crustaceans (Riley 1986). The pentastomid genus *Armillifer* normally inhabits the respiratory tracts of snakes, although larval-stages (nymphs) of these parasites can infect humans leading to a medical condition known as pentastomiasis (Tappe & Büttner 2009). Since pentastomid nymphs are difficult to identify morphologically, we aimed here to sequence the complete mitogenome of *Armillifer grandis* – an African species known to infect humans (Fain & Salvo 1966) – to facilitate the molecular identification of both adult and/or juvenile instars. In a wider context, we wish to promote the still rather preliminary genetic studies of pentastomids.

An adult *Armillifer grandis* specimen was collected from the lungs of a rhinoceros viper (*Bitis nasicornis*) in the Democratic Republic of Congo near the town of Kole (3°27′ 37′′N, 22°26′′′E, Sankuru district) and identified morphologically to species level (Tappe et al. 2016). A voucher is deposited in the pentastomid collection of the Museum für Naturkunde Berlin (Röhlig et al. 2010) under the accession ZMB 48784. The complete mitogenome was obtained using next-generation shotgun sequencing. Paired end Illumina sequencing libraries were generated from the tissue sample and sequenced on an Illumina NextSeq500 platform, using Illumina NextSeq^®^ 500/550 High Output Kit V2. Sequencing yielded 3,452,321 of 150 bp paired end reads. A complete circularized mitochondrial genome was obtained with NOVOplasty 2.4 (Dierckxsens et al. 2017) using kmer 47, and the mitogenome of *Armillifer agkistrodontis* (NC_032061) as a bait reference. The assembled mitogenome was manually inspected for repeats at the transcript ends to confirm circularity. Annotations were carried out with MITOchondrial genome annotation Server (MITOS) (Bernt et al. 2013), and manual validation of the coding regions using the NCBI ORF Finder (http://www.ncbi.nlm.nih.gov/gorf/gorf.html) in combination with NCBI’s Conserved Domain Database (CDD) (Marchler-Bauer et al. 2017). The annotated sequence file was submitted to NCBI (accession no. KY914472). The phylogenetic position of the new sequence of *Armillifer grandis* according to the gene Cytochrome oxidase I is shown in Figure 1. As expected, it resolves among other *Armillifer* taxa closest to another African species: *Armillifer armillatus*.

**Figure 1.**
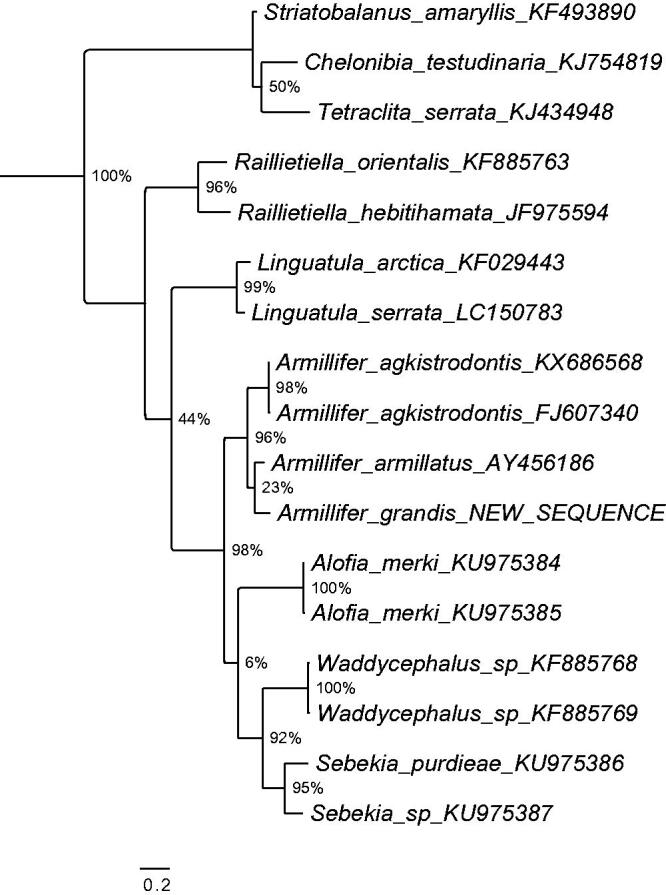
Maximum likelihood tree illustrating the phylogenetic position of the newly sequenced *Armillifer grandis* gene sequence among a subset of pentastomid species. Cytochrome oxidase I sequences were aligned using MAFFT 7.271 and highly divergent or poorly aligned regions were removed with Gblocks 0.91b (Castresana 2000) allowing for gap positions and smaller blocks. Trees were calculated using PhyML 3.1 (Guindon et al. 2010) with 12 rate categories, optimized equilibrium frequencies, GTR model of sequence evolution and combined heuristics (Nearest Neighbor Interchange and Subtree Pruning and Rerafting). Branch support was calculated using approximate likelihood ratio tests as implemented in PhyML.

The complete mitochondrial transcript of *Armillifer grandis* was 16,073 bp in length and contained 13 protein-coding genes (PCGs), 2 ribosomal RNA genes, and 22 transfer RNA genes. As described for other pentastomid mitogenomes (Lavrov et al. 2004; Li et al. 2016), the mitochondrial genome of *Armillifer grandis* contained an A + T bias with an overall base composition of 34.6% A, 29.4% T, 29% C, and 6,9% G. The gene arrangement of the present mitogenome is identical to those of other pentastomids investigated so far (Lavrov et al. 2004; Li et al. 2016).

Most of the genes are encoded on the L-strand with the exception of four protein-coding genes (*NAD5*, *NAD4*, *NAD4L*, *NAD1*), nine tRNA (*tRNA^Cys^*, *tRNA^Gln^*, *tRNA^Tyr^*, *tRNA^Phe^*, *tRNA^His^*, *tRNA^Thr^*, *tRNA^Pr^°*, *tRNA^Val^*, *tRNA^LeuCUN^*), and both rRNAs (12S and 16S), which were encoded in the H-strand. Six PCGs (*ND2*, *COX2*, *ATP8*, *ATP6*, *ND3* and *CYTB*) had ATA as the initiation codon, while five PCGs (*COX3*, *ND5*, *ND4*, *ND4L* and *ND1*) presented ATG as the initiation codon. An alternative initiation codon CTG was found for *COX1*, and an ATC initiation codon for *ND6*. Incomplete termination codons were found for five PCGs (*COX1*, *COX2*, *COX3*, *ND5* and *ND4*) which are complemented with the addition of 3′A residues. Six PCGs (*ATP8*, *ATP6*, *ND3*, *ND6*, *CYTB and ND1*) used a TAA termination codon, while *ND4L* used a TAG termination codon. The *12S* and *16S* genes had a length of 656 and 1145 bp, respectively.
